# Stable Gastric Pentadecapeptide BPC 157—Possible Novel Therapy of Glaucoma and Other Ocular Conditions

**DOI:** 10.3390/ph16071052

**Published:** 2023-07-24

**Authors:** Predrag Sikiric, Antonio Kokot, Tamara Kralj, Mirna Zlatar, Sanja Masnec, Ratimir Lazic, Kristina Loncaric, Katarina Oroz, Marko Sablic, Marta Boljesic, Marko Antunovic, Suncana Sikiric, Sanja Strbe, Vasilije Stambolija, Lidija Beketic Oreskovic, Ivana Kavelj, Luka Novosel, Slavica Zubcic, Ivan Krezic, Anita Skrtic, Ivana Jurjevic, Alenka Boban Blagaic, Sven Seiwerth, Mario Staresinic

**Affiliations:** 1 Department of Pharmacology, School of Medicine, University of Zagreb, 10000 Zagreb, Croatia; tamara_kralj@yahoo.com (T.K.); mirnazlatar@yahoo.com (M.Z.); sanjamp@yahoo.com (S.M.); rratimir@yahoo.com (R.L.); kristinaloncaric@gmail.com (K.L.); oroz.kat@hotmail.com (K.O.); mark.antun@gmail.com (M.A.); strbes@gmail.com (S.S.); vasilije.stambolija@gmail.com (V.S.); lidijabeketicoreskovic@gmail.com (L.B.O.); ivana.kavelj@gmail.com (I.K.); novosel0701@gmail.com (L.N.); slavica.zubcic.kristo@bolnica-zadar.hr (S.Z.); ivankrezic94@gmail.com (I.K.); ivana.jurjevic@mef.hr (I.J.); abblagaic@mef.hr (A.B.B.); 2 Department of Anatomy and Neuroscience, Faculty of Medicine, J.J. Strossmayer University of Osijek, 31000 Osijek, Croatia; marko.sablic@mefos.hr (M.S.); mboljesic@mefos.hr (M.B.); 3 Department of Pathology, School of Medicine, University of Zagreb, 10000 Zagreb, Croatia; suncanasikiric@gmail.com (S.S.); sven.seiwerth@mef.hr (S.S.); 4 Department of Surgery, School of Medicine, University of Zagreb, 10000 Zagreb, Croatia; ravnateljstvo@kb-merkur.hr

**Keywords:** BPC 157, glaucomatous rats, intraocular pressure, retinal integrity, pupil function, retinal ischemia, corneal injuries, maintained transparency, therapy

## Abstract

Recently, stable gastric pentadecapeptide BPC 157 therapy by activation of collateral pathways counteracted various occlusion/occlusion-like syndromes, vascular, and multiorgan failure, and blood pressure disturbances in rats with permanent major vessel occlusion and similar procedures disabling endothelium function. Thereby, we revealed BPC 157 cytoprotective therapy with strong vascular rescuing capabilities in glaucoma therapy. With these capabilities, BPC 157 therapy can recover glaucomatous rats, normalize intraocular pressure, maintain retinal integrity, recover pupil function, recover retinal ischemia, and corneal injuries (i.e., maintained transparency after complete corneal abrasion, corneal ulceration, and counteracted dry eye after lacrimal gland removal or corneal insensitivity). The most important point is that in glaucomatous rats (three of four episcleral veins cauterized) with high intraocular pressure, all BPC 157 regimens immediately normalized intraocular pressure. BPC 157-treated rats exhibited normal pupil diameter, microscopically well-preserved ganglion cells and optic nerve presentation, normal fundus presentation, nor- mal retinal and choroidal blood vessel presentation, and normal optic nerve presentation. The one episcleral vein rapidly upgraded to accomplish all functions in glaucomatous rats may correspond with occlusion/occlusion-like syndromes of the activated rescuing collateral pathway (azygos vein direct blood flow delivery). Normalized intraocular pressure in glaucomatous rats corresponded to the counteracted intra-cranial (superior sagittal sinus), portal, and caval hypertension, and aortal hypotension in occlusion/occlusion-like syndromes, were all attenuated/eliminated by BPC 157 therapy. Furthermore, given in other eye disturbances (i.e., retinal ischemia), BPC 157 instantly breaks a noxious chain of events, both at an early stage and an already advanced stage. Thus, we further advocate BPC 157 as a therapeutic agent in ocular disease.

## 1. Introduction

We suggest stable gastric pentadecapeptide BPC 157 in glaucoma therapy. It may be relevant as a potent cytoprotective agent with strong vascular rescuing capabilities [1,2,3,4,5,6,7,8,9,10] as a novel cytoprotective approach in glaucoma therapy [11]. This was noted in counteraction of rat occlusion/occlusion-like syndromes as an activation of the collateral-rescuing vascular pathway [1]. These occurred during permanent occlusion of major vessels peripherally and centrally (including episcleral vein cauterization to induce glaucoma) or similar noxious procedures that all severely disable endothelium function [1].

### 1.1. Particular Cytoprotective Aspects of BPC 157 Therapy in Glaucoma

This review attempts to reveal these particular aspects of BPC 157 therapy that may also be useful in the therapy of glaucoma. It was shown to promptly recover glaucomatous rats [11] and rapidly normalize intraocular pressure [11]. It maintained retinal integrity [11], recovered pupil function [11,12], and recovered retinal ischemia [13]. Furthermore, it recovered corneal injuries and recovered corneal transparency [14,15,16,17]. Recovered corneal injuries and recovered corneal transparency were exemplified in rats who underwent complete corneal abrasion [14], corneal ulceration [15], lacrimal gland removal dry eye, or corneal insensitivity [16,17]. In general, the BPC 157 therapy highlights its particular capabilities, cytoprotection [7] (i.e., translation of the original protection of stomach epithelial cells to the protection of other organs (organoprotection)) and consequent particular vascular effect [1], wound healing [8], and neuroprotection [9]. Based on the evidenced beneficial effects, its particular role in the functioning of the brain–gut and gut–brain axes is recently fully emphasized [10]. In conclusion, it might be the cytoprotection phenomenon with application in distinctive injuries, analogous to, if not identical, that might occur during major vessel occlusion and application of other similar noxious procedures [18,19,20,21,22,23,24,25,26,27,28,29,30]. Furthermore, it might be that in the cytoprotection cases, the same agent—pentadecapeptide BPC 157, also suggested being the novel native and stable cytoprotection mediator in human gastric juice, for more than 24 h, and easily applicable [1,2,3,4,5,6,7,8,9,10,11]—might be responsible for the effect.

### 1.2. The Proposed Cytoprotective Principle vs. Pitfalls of Standard Intraocular Pressure-Lowering Drugs

On the other hand, the proposed cytoprotective principle of BPC 157 therapy in glaucoma [11] may overwhelm principles commonly acknowledged for potent and efficacious intraocular pressure-lowering drugs. Namely, the antiglaucoma therapy course and development may be illustrative since it was vividly described (1876, Ludwig Laqueur) in many reviews [31,32,33,34]. Evidently, there is no common concept. Frequently, the intraocular pressure lowering agents showed discordant effectiveness. Some may be effective only in systemic but not topical applications (propranolol, acetazolamide) [34]. The intraocular pressure lowering agents constitute very distinctive classes that were also regularly combined, since very early times (i.e., pilocarpine and epinephrine [31,32,33,34]). Particular targets appeared for the beta-blockers, carbonic anhydrase inhibitors, alpha-adrenergic agonists (aqueous humor inflow inhibition) [35,36,37,38,39], muscarinic agonists, and rho kinase inhibitors (aqueous humor outflow stimulation) [36,37,38,39]. All were thought to be revolutionized by the prostaglandin issue [40]. As an important indicative point, prostaglandins were introduced at that time as the first mediators of cytoprotection. Cytoprotection with prostaglandins was introduced as a concept of general (healing) significance. This was to directly prevent epithelial necrosis that may arise in the stomach from the direct injurious effect of various agents’ applications, and thereby in other tissues as well [41,42,43,44,45,46,47,48,49,50]. Of note, in initial [51,52,53,54,55] and later [56,57,58,59] eye studies, these points (i.e., pleiotropic cytoprotective beneficial effects of prostaglandins in glaucoma therapy) were not combined. The medical treatment following the approved formulation of latanoprost [60] resulted in annual global sales of more than 1 billion U.S. dollars [34]. Naturally, these implied resolutions (lower doses) [54,55,56,57,58,59] of severe ocular inflammation, resulted in ocular hypertension, pupillary miosis, and breakdown of the blood–aqueous barrier by prostaglandins (high doses) [51,52,53]. In addition, conceptual pitfalls regularly appeared in anti-glaucoma therapy courses and development. With miotics, this was since the initial introduction of physostigmine and pilocarpine application [61,62], which was the mainstay of glaucoma treatment for 100 years, and is still in use [31,32,33,34]. This was the paradoxical effect, a net rise of intraocular pressure, and impaired uveoscleral outflow in the condition of the severely compromised and unresponsive trabecular outflow [63]. Beta-blockers started with propranolol. Pitfalls encountered were intravenous applications lowering intraocular pressure, avoiding corneal anesthetic properties, negative effect on tear production, profound dry eyes syndrome, subconjunctival fibrosis, and tachyphylaxis [64]. It ended with the development of topical timolol [31,32,33,34,64,65]. However, it characterized the paradox of timolol concomitant to epinephrine to enhance the ocular hypotensive effects in many patients [66]. Additionally, with the maximal clinical effect of timolol on increased intraocular pressure, minimal bounding to beta receptors occurred [67]. In addition, patients responding to beta-agonists with a reduction in intraocular pressure may have a decrease in intraocular pressure when timolol is administered locally [66]. Likewise, to avoid unpleasant side effects of systemic carbonic anhydrase inhibition, carbonic anhydrase inhibitors, since acetazolamide (topical administration with little or no effect on intraocular pressure) [68,69], were used for a long period before a topical carbonic anhydrase inhibitor was realized (dorzolamide) [70,71].

### 1.3. The Proposed BPC 157 Cytoprotective Principle

Thus, with respect to the known therapy, achievement, and pitfalls [31,32,33,34], the proposed BPC 157 cytoprotective principle [1,2,3,4,5,6,7,8,9,10] should be viewed. As emphasized, the cytoprotective arguments are recovery of glaucomatous rats [11], normalized intraocular pressure [11], maintaining retinal integrity [11], recovering pupil function [11,12], recovering retinal ischemia [13], and corneal injuries [14,15,16,17]. In particular, counteracting corneal drying, counteracting the loss of corneal sensation, counteracting decrease in blink rate, and maintaining tear production [16,17], have equal effectiveness for topical and systemic application. Thereby, the proposed BPC 157 cytoprotective principle should be more viable for further eye therapy.

Consequently, although far from the established clinical evidence [11], the important BPC 157 argument not implied in glaucoma therapy so far, might be that BPC 157 therapy has a wide cytoprotection agenda [1,2,3,4,5,6,7,8,9,10] that may be advantageous for eye pharmacotherapy. Hence, the additional important BPC 157 argument is its long stability in human gastric juice (i.e., 24 h), and thereby, its applicability for therapy as a native cytoprotection mediator mediating gastrointestinal mucosal integrity [1,2,3,4,5,6,7,8,9,10]. Note, the cytoprotection concept made by Robert (prevention of stomach epithelial cell necrosis) [41,42,43,44] and Szabo (endothelium protection) [45,46,47,48,49,50] links the original protection of stomach epithelial cells to the protection of other organs (organoprotection) [42,47] (i.e., for eye pharmacotherapy, stomach epithelium behaves like eye epithelium [15]), and stomach-cytoprotective agents to agents with pleiotropic beneficial effect [42,47]. Thereby, BPC 157 is easily applicable and safe without adverse effects (i.e., in ulcerative colitis trials) while toxicology studies showed that a lethal dose (LD1) can be not achieved (for review, see [1,2,3,4,5,6,7,8,9,10]). Consequently, we used both local and systemic applications (including via the per-oral way) [1,2,3,4,5,6,7,8,9,10].

#### The Proposed BPC 157 Cytoprotective Principle as the Counteraction of the Occlusion/Occlusion-like Syndromes

Recently, regarding the particular point of resolving the application in eye pharmacotherapy, the pleiotropic beneficial effect as a particular cytoprotective agent’s ability was combined with the cytoprotective ability of the pentadecapeptide BPC 157 to further maintain and upgrade endothelium integrity and functioning (for review see [1,2,3,4,5,6,7,8,9,10]). In particular, the counteraction of the occlusion/occlusion-like syndromes consistently evidenced in studies [18,19,20,21,22,23,24,25,26,27,28,29,30], focused minor vessels (i.e., activated azygos vein to direct blood flow delivery) and rapid upgrading to substitute the function of the failed major blood vessels. By doing so, activation of the collateral pathways, “bypassing vascular key” depending on the given injury, may occur as a particular consequent effect of the therapy [18,19,20,21,22,23,24,25,26,27,28,29,30]. Commonly, there was the recovery of the severe occlusion/occlusion-like syndrome, the recovery of multiorgan failure syndrome in the rats with vascular failure induced by major vessels’ occlusion, peripherally [18,19,20,21,22,23] and centrally [18,19,20,21,22,23,24,25,26,27,28,29,30], and other similar noxious procedures [18,19,20,21,22,23,24,25,26,27,28,29,30] that largely affect endothelium function, and multicausal pathology was fully recovered [18,19,20,21,22,23,24,25,26,27,28,29,30]. Thereby, such recovery of the occlusion/occlusion-like syndrome may be relevant [18,19,20,21,22,23,24,25,26,27,28,29,30]. This may be of particular relevance for the recovery of glaucoma (episcleral vein cauterization) [11] (i.e., rapid decrease in the increased intraocular pressure and preserved retinal integrity) [11], along with the recovery of pupil function [12], retinal ischemia [13], and corneal injuries (i.e., those induced by complete corneal abrasion [14], corneal ulcer [15], or dry eye [16,17]). As an example of the tightly interconnected lesions that multiorgan failure causes, there may be a wide interconnected therapy effect in the recovery of the occlusion/occlusion-like syndrome [18,19,20,21,22,23,24,25,26,27,28,29,30]. There were general blood pressure disturbances (i.e., intracranial (superior sagittal sinus), portal and caval hypertension, and aortal hypotension). The lesions occurred in the brain (including intracerebral and intraventricular hemorrhage), heart (severe arrhythmias, congestion, and endocardial infarction), lungs (hemorrhage), and congestion in the liver, kidney, and gastrointestinal lesions. Major vessels were congested (i.e., inferior caval vein, superior mesenteric vein), azygos vein collapsed, venous and arterial thrombosis progressed, peripherally and centrally, and advanced Virchow triad was fully substantiated. These were all attenuated/eliminated by BPC 157 therapy [18,19,20,21,22,23,24,25,26,27,28,29,30]. Illustratively, major vessel congestion was reversed to normal vessel presentation, the recovered azygos vein reactivated the pathway for direct blood flow delivery, and the vascular failure (and Virchow triad circumstances) was effectively cured [18,19,20,21,22,23,24,25,26,27,28,29,30].

### 1.4. Cytoprotection Agenda in Glaucoma Therapy Distinctive from the Focused Background of the Beta-Blockers, Alpha 2-Agonists, Inhibitors of Carbonic Anhydrase, or Parasympathomimetics, and Prostaglandin Derivatives

Such a wide cytoprotection agenda in glaucoma therapy might also be distinctive from the focused background of the beta-blockers, alpha 2-agonists, inhibitors of carbonic anhydrase, or parasympathomimetics, and prostaglandin derivatives. Illustratively, susceptible to multiple types of toxicity, corneal endothelium in patients with glaucoma may incur damage, also due to various medical and surgical interventions in addition to the disease itself [72,73]. With glaucoma pharmacotherapy reducing intraocular pressure, there is a common concern given to the potential impact of these drugs on the corneal endothelium [74,75]. Furthermore, a decrease in the number and density of corneal subbasal nerve fiber bundles commonly appeared consequent to chronic administration of glaucoma medications [76]. Illustratively, beta blockers inhibit corneal reepithelization [76,77,78], and clonidine, as an alpha2-adrenoreceptor agonist, might damage the cornea and impair human vision [79]. Likewise, brinzolamide may induce corneal edema [80,81]. Topical carbonic anhydrase inhibitors can cause damage to corneas with severe endothelial dysfunction [82,83,84,85]. Several studies indicated that topical anti-glaucoma therapy with prostaglandin analogs induced a significant decrease in central corneal thickness, as well as in patients with normal tension glaucoma [86,87,88,89]. Of note, there may be similar indicative practical limitations for the prostaglandins cytoprotection concept [42,43,44,45,46,47,48,49,50], although the cytoprotection concept is still considered to be one of the major theoretical breakthroughs of how epithelium and endothelium function maintenance can be maintained [7]. In addition, there was limited efficacy, and a lacking of curative effects in the original cytoprotection implementation by prostaglandins given (pleiotropic) beneficial effects occurring mostly with application before injury induction [42]. On the contrary, parasympathomimetics facilitate corneal wound healing [90,91], and rho kinase inhibitor enhances wound healing in the corneal endothelium [92,93]. 

Thus, as opposed to the present discrepancy, there may likely be a relevant resolution with the consistent anti-glaucomatous effect of the pentadecapeptide BPC 157 based on its beneficial cytoprotective effect.

### 1.5. BPC 157 Therapy, Interaction with Essential Systems, i.e., Nitric Oxide (NO), Prostaglandins-System, Implicated in the Cytoprotection Concept and Glaucoma

For BPC 157 therapy, a common successful result might be the combined evidence, involving essential systems, i.e., nitric oxide (NO)-, prostaglandins-system, long-ago implicated in the cytoprotection concept [1,2,3,4,5,6,7,8,9,10]. These systems are also commonly acknowledged as essential in glaucoma [94,95]. This would explain the particular vascular effect and recovery [1,2,3,4,5,6,7,8,9,10] that may take part in eye and glaucoma therapy, as evidenced by particular effects on NO-agents in eye studies [11,12,13,14,15,16,17]. As mentioned, it might be the combined chain of tightly interconnected subsequent events. The maintained endothelium function (for review see [1,2,3,4,5,6,7,8,9,10]) had long ago been recognized as an immediate part of the innate activity of the cytoprotective agent [46,47,48,49,50]. Thereby, BPC 157 induced NO-release of its own [96,97,98,99]. Then, the BPC 157 activation of the collateral pathways, “bypassing vascular key” [18,19,20,21,22,23,24,25,26,27,28,29,30], the induced NO-release of its own [96,97,98,99], occurred with the entire NO-system as the interaction or the modulation. This modulation to maintain and reestablish normal system functioning may occur as the counteraction of the NO-synthase blockade (N(G)-nitro-L-arginine methyl ester (L-NAME)-hypertension, and pro-thrombotic effect counteracted). In eye pharmacotherapy, there was a prompt counteraction of the retinal ischemia induced by the retrobulbar application of L-NAME [13]. Likewise, maintaining modulation and reestablishing normal system functioning may occur as the counteraction of NO-synthase (NOS)-substrate over-activity (L-arginine-hypotension and anti-thrombotic effect counteracted) [98,100]. Thus, both vessel and thrombocyte functions are specifically allocated. The coagulation pathways were not affected (aggregometry and thromboelastometry studies) [100,101,102]. The VEGFR2-Akt-eNOS signaling pathway was directly activated (i.e., without the need for other known ligands or shear stress), controlling vasomotor tone (both smooth muscle and endothelium), and the Src-Caveolin-1-eNOS pathway was activated [103,104]. These occurred along with the rapid change in the lipid contents and protein secondary structure conformation produced in the vessel wall by BPC 157 therapy (Fourier transform infrared spectroscopy [105]). Simultaneous modulatory effects on the prostaglandins system can be clearly envisaged by counteraction of NSAIDs toxicity (peripheral and central organ lesions, bleeding, thrombocytopenias [100,101,102,106], and in particular, leaky gut syndrome) [107] and interaction with many molecular pathways [103,104,105,107,108,109,110,111,112,113,114,115]. Thereby, the rapid and then sustained outcome is functionally improved minor vessels, successfully taking the function of disabled major vessels, reestablishing reorganized blood flow and functioning even in the worst circumstances [18,19,20,21,22,23,24,25,26,27,28,29,30].

### 1.6. BPC 157 and Glaucoma Therapy

Thus, from this point, our glaucoma studies start [11]. Glaucomatous rats presented with severe retinal damage and failed function were rats with permanent venous congestion and permanently increased intraocular pressure induced by cauterization of three of four episcleral veins (open-angle glaucoma model) [116,117,118,119,120,121]. Regularly, glaucoma-like features are constant. Namely, without upgrading, one sole episcleral vein function could not perform the whole function of aqueous fluid drainage [116,117,118,119,120,121]. Of note, the cauterization of three of four episcleral veins [11] may be a specific challenge for this novel therapeutic approach with stable gastric pentadecapeptide BPC 157. The specific challenge that was resolved, was the activation of the collateral pathways and upgrading of the particular minor vessel (i.e., remaining episcleral vein that was not cauterized) [11]. This occurred as an adequate translation of the function of the failed major vessels (the three cauterized episcleral vessels), thereby, reestablishing the reorganized blood flow and function recovery. This chain of events consistently occurred in the recovery of the occlusion/occlusion-like syndrome [18,19,20,21,22,23,24,25,26,27,28,29,30], and it was shown to also be effective toward this specific target, namely, the remaining episcleral vein [11]. Consequently, the anti-glaucomatous effect of the stable gastric pentadecapeptide BPC 157 therapy (for review, see [1,2,3,4,5,6,7,8,9,10]) may be prompt vascular recovery, specifically related to the remaining episcleral vein as a notable therapy target [11].

## 2. Glaucoma

### 2.1. Model Consideration

There is a close matching of uveoscleral outflow pathways and aqueous dynamics in rats and humans. Likewise, there are general ocular anatomical structure similarities [121,122,123,124]. Thus, the fulfillment of essential requests (three cauterized episcleral veins) necessitate that a new concept and therapy introduction may be relevant for further pharmacology studies. In principle, these should be helpful to analyze BPC 157 therapy application in glaucoma [11].

Suited for glaucoma research, cauterization of two episcleral veins may produce a considerable elevation of intraocular pressure. Likewise, the cauterization of two episcleral veins may produce marked retinal ganglion cell damage [125,126]. Thereby, cauterization of three of four episcleral veins, more commonly used (i.e., [11,127,128,129,130,131]), provides a more severe glaucoma course. As such, it regularly produces inescapable venous congestion, increased intraocular pressure (intraocular pressure >2 times that of normal pressure [11,131]), and consequent injurious course in rats. As leading symptoms that should be counteracted, rats continuously expressed increased intraocular pressure and mydriasis. Furthermore, they exhibited degeneration of retinal ganglion cells, optic nerve head excavation and reduction in optic nerve thickness, generalized severe irregularity of retinal vessels, faint presentation of choroidal vessels, and severe optic nerve disc atrophy. The six-week period used seems to be suited for adequate model elaboration [11].

### 2.2. BPC 157 Therapy May Rapidly Induce Normalization of Intraocular Pressure

Consequently, the BPC 157 therapy anti-glaucomatous effect is a particularly beneficial effect. Moreover, topical, per-oral, and intraperitoneal application of the stable gastric pentadecapeptide BPC 157 therapy may rapidly induce normalization of intraocular pressure. Once applied, it lasts for at least 24 h [11] and is sustainably maintained in the long-term with daily administration. This equipotent high efficacy may have a prophylactic effect as application immediately before surgery to avoid glaucoma course development. Likewise, the equipotent high efficacy is of therapeutic significance as a delayed regimen. Reversal of already advanced course and normalization of established elevated intraocular pressure may rapidly appear. Thus, both prophylactic and therapeutic potential is evident. The remaining episcleral vein was promptly upgraded to venous congestion counteraction. This occurred regardless of whether three episcleral veins had been cauterized, and remained permanently non-functioning. As a rapid and sustained effect (and final suited outcome), the raised intraocular pressure was avoided/counteracted providing the application time was before induction or in the already advanced course.

In practice, this resolved the otherwise insurmountable glaucoma course, i.e., both prevention and reversal of an advanced elevated intraocular pressure course. The rapid onset of the effect, i.e., normalization of the elevated intraocular pressure, is quite consistent and fully supported in occlusion/occlusion-like syndromes research [18,19,20,21,22,23,24,25,26,27,28,29,30]. Such a very rapid effect occurred as in the previous occlusion/occlusion-like syndromes during permanent major vessel occlusion, peripheral and central, and similar procedure applications related to considerable endothelium dysfunction [18,19,20,21,22,23,24,25,26,27,28,29,30]. Additionally, such an effect means that there is a consistent matching of pressure disturbances, i.e., intraocular (intraocular pressure) and extra-ocular (intracranial, portal, caval hypertension, and aortal hypotension). This may have a more general significance. The rapid upgrading of the various collateral pathways [18,19,20,21,22,23,24,25,26,27,28,29,30] may have applicability in various vessel tributaries [18,19,20,21,22,23,24,25,26,27,28,29,30] and an additional beneficial effect, including the eye specifically [11]. Thereby, the normalizations of the glaucoma disturbances [11] may be also similar to the normalization/attenuation of intracranial (sinus sagittal) hypertension, portal, and caval hypertension, and aortal hypotension. Thus, the anti-glaucoma effect may be combined with counteraction of the vessel and multiorgan failure syndrome, peripherally and centrally, in occlusion/occlusion-like syndromes [18,19,20,21,22,23,24,25,26,27,28,29,30].

### 2.3. BPC 157 Therapy Exhibited Immediate Normalization of Elevated Intraocular Pressure as a General Beneficial Effect

Such rapid normalization of elevated intraocular pressure by BPC 157 therapy appears as a generally beneficial effect on other leading symptoms that should all be counteracted. As the most important point, this should also be suited for further therapy application. Namely, rats continuously expressed full glaucoma syndrome. They exhibited increased intraocular pressure and mydriasis, degeneration of retinal ganglion cells, optic nerve head excavation and reduction in optic nerve thickness, generalized severe irregularity of retinal vessels, faint presentation of choroidal vessels, and severe optic nerve disc atrophy. As a general tightly interconnected effect, these were all counteracted by BPC 157 therapy [11]. In this, there was consistent efficacy of the daily regimens supporting each other’s efficacy. Ascertaining both local and systemic application (including the most convenient per-oral) means that topical corresponded to per-oral and intraperitoneal applications. Topical dosage was 0.4 µg/eye to 0.4 ng/eye. Per-oral was in drinking water (0.16 µg/mL to 0.16 ng/mL, 12 mL/rat until sacrifice), the first application being intragastric. The intraperitoneal application was the last application at 24 h before sacrifice. They all shared the same wide dose range of 10 µg/kg to 10 ng/kg, and high therapy range [11]. All BPC 157-treated rats exhibited normal intraocular pressure and normal pupil diameter. Microscopically and fundoscopically, they exhibited well-preserved ganglion cells and optic nerve presentation, normal fundus presentation, normal retinal and choroidal blood vessel presentation, and normal optic nerve presentation [11]. Thus, analogous with the therapy effects noted in the counteraction of the severe occlusion/occlusion-like syndromes [18,19,20,21,22,23,24,25,26,27,28,29,30], such consistent results with a correspondingly wide range of the agent’s efficacy (µg–ng) support each other for further therapy use [11]. Additionally, the ophthalmic vein can also serve as a rescuing pathway in rats with central vein (superior sagittal sinus) occlusion [24]. The bypassing loop was along the angular vein, facial anterior and posterior vein, facial vein, via the external jugular vein, and subclavian vein, through the superior caval vein. Consequently, analogous with glaucoma-induced by three episcleral vein cauterizations and elevated intraocular pressure, and rapid counteraction, the brain swelling rapidly attenuated, and the intracranial hypertension (i.e., increased pressure in the ligated superior sagittal sinus) was promptly eliminated [24].

Furthermore, these may be particular effects [11] in many respects (see below).

### 2.4. Retinal Ischemia

First, retinal ischemia as the final drawback [118,119,120,121] was likely to be resolved [11]. Previously, BPC 157 application annihilated that, which the L-NAME retrobulbar administration would otherwise rapidly induce [14] (see Section 4, Retinal Ischemia). Thereby, also for anti-glaucoma therapy with essential NO-importance [94], it may be quite indicative of the counteraction of the NO-synthase (NOS)-blocker effect, in particular, and thereby, counteraction of NO-system failure (i.e., retinal ischemia) supposedly particularly damaging for eye function [94,132,133,134,135,136]. This can be ascribed to its modulatory effects on the NO-system [96,97,98,99,100] and prostaglandins-system [106,107], as mentioned above. As emphasized, although not specifically investigated in the glaucoma rats, the recently described activation of the Src-Caveolin-1-eNOS pathway [103,104] and interaction with several molecular pathways [103,104,107,108,109,110,111,112,113,114,115] may be important for maintaining vasomotor tone, and thereby essential for prompt therapeutic effect in glaucoma rats. In addition, BPC 157 may counteract the disturbances of gap junction [107] postulated in cell death and neuromodulation in the retina [137] as BPC 157 acts as a stabilizer of the cellular junction [107]. Namely, BPC 157 mitigated leaky gut syndrome by acting via increasing tight junction protein ZO-1 expression and transepithelial resistance [107]. Additionally, there was inhibition of mRNA of inflammatory mediators (iNOS, IL-6, IFN, and TNF-alpha), while BPC 157 increased expression of HSP 70 and 90, and antioxidant proteins, such as HO-1, NQO-1, glutathione reductase, glutathione peroxidase 2, and GSTpi [107].

### 2.5. Retina–Brain Axis, Brain–Gut Axis, Gut–Brain Axis

Furthermore, along with the vascular recovery background of the BPC 157 anti-glaucoma therapy [11], and the very high extent of the recovery achieved by the therapy, an important part of its anti-glaucoma therapy effect may also be BPC 157 participation in the retina–brain axis. Namely, the retina and the brain share many supporting functional and structural similarities [138], and as one of the longest central nerves, the optic nerve connects the retina to the thalamic brain nuclei [31]. This may be likely, since BPC 157 based on the reported simultaneous effects in the brain and periphery may mediate the gut–brain and brain–gut axis [1,2,3,4,5,6,7,8,9,10]. There is a very high extent of recovery in the brain, noted in stroke rats, and with occlusion/occlusion-like syndrome [18,19,20,21,22,23,24,25,26,27,28,29,30]. These were rats having permanent major vessel occlusion, peripherally [18,19,20,21,22] and centrally [24], or with other similar noxious procedures applied [26,27,28,29,30], which severely affect endothelium function. In stroke-rats, both early and delayed neural hippocampal damages were counteracted and debilitated functions were completely recovered after reperfusion. In addition, in occlusion/occlusion-like syndromes, brain swelling and lesions, intracerebral and intraventricular hemorrhage, and intracranial (superior sagittal sinus) hypertension were counteracted. In the periphery, portal and caval hypertension, aortal hypotension, and lesions and hemorrhage in the internal organs were counteracted. Progressing thrombosis, in the artery and vein, was counteracted peripherally and centrally. Virchow triad circumstances were counteracted in general [18,19,20,21,22,23,24,25,26,27,28,29,30] (thrombocytes function recovered, without interference with coagulation [100,101,102]). Particularly in vascular occlusion studies [18,19,20,21,22,23,24,25,26,27,28,29,30], BPC 157 was found as a free radical scavenger [107,108,139,140,141,142,143,144,145] along with its action as a stabilizer of cellular junctions [108]. This may emphasize the relevance of the counteraction by BPC 157 therapy of progressive severe atrophy of the optic nerve in rats with the normal intraocular pressure [13] that was otherwise regularly induced by retrobulbar L-NAME application. The neuroprotective effect of BPC 157 administration may also be responsible for the counteraction of the retinal disturbances in glaucomatous disturbances, even with normal intraocular pressure [11,13]. Noteworthy, normotension glaucoma has poor retinal blood flow [31,146].

### 2.6. Mydriasis

Second, there are also established mydriasis relations to intraocular pressure in humans [147,148,149]. These can likely be relevant to envisage the possible translational significance of maintaining pupillary function by BPC 157 [11,12]. Illustratively, latanoprost caused miosis, with rebound mydriasis at 24 h post-treatment [150]. Timolol has a delayed effect on miosis (from 4 to 8 h post-treatment), as well as in normal eyes [151,152]. Dorzolamide had no effect [153]. Pilocarpine induced 30 min–12 h miosis in normal eyes [154]. Contrarily, since BPC 157 does not affect normal intraocular pressure [11,12], nor normal pupil diameter [12], mydriasis↔elevated intraocular pressure and thereby therapy, seems to be present with BPC 157 therapy (topical, per-oral, intraperitoneal application, equipotent high efficacy). There were indicative counteractions of both atropine-induced mydriasis and glaucoma-induced elevated intraocular pressure and mydriasis, in parallel, as a rapid and sustained effect [11,12]. Thereby, with BPC 157 therapy, vascular recovery means eliminating blocking drainage of the intraocular fluid from the angle of the anterior chamber in the dilated iris as a particular and parallel effect on recovering normal intraocular pressure in glaucomatous rats [11,12]. Additionally, BPC 157 consistently recovered smooth muscle function, in particular, various sphincters [2,6,10]. Such clear matching may be an advantageous effect, a general healing effect over both the ambiguous pressure-lowering effect and mydriasis counteraction effect of the standard anti-glaucoma agents. Importantly, the standard anti-glaucomatous agents on the increased intraocular pressure had delayed onset [155,156,157,158,159,160,161,162], and in some experimental studies, the effect can be absent [163], or even the opposite effect [164].

### 2.7. Consistent Therapy Findings on Both Injured Eyes

Finally, we should emphasize the significance of bilateral studies. In addition, counteraction of the more severe course may be more reliable for primary open-angle glaucoma as a bilateral progressive chronic optic neuropathy [165,166,167,168]. A particular consistency of the BPC 157 eye therapy occurred in both injured eyes and their injuries are equally counteracted [11,12,13,14,15,16,17]. In such bilateral studies, the consistent therapy findings on both injured eyes may be a particular confirmation of the applicability of the effect.

Contrarily, the findings of only one experimental eye with less severe circumstances [125,126,127,128,129,130,131] may have more limitations. Namely, neuronal degeneration and glial activation [168,169,170,171,172,173] also affect fellow control eyes making unilateral models of retinal injury less completely defined.

**Figure 1:**
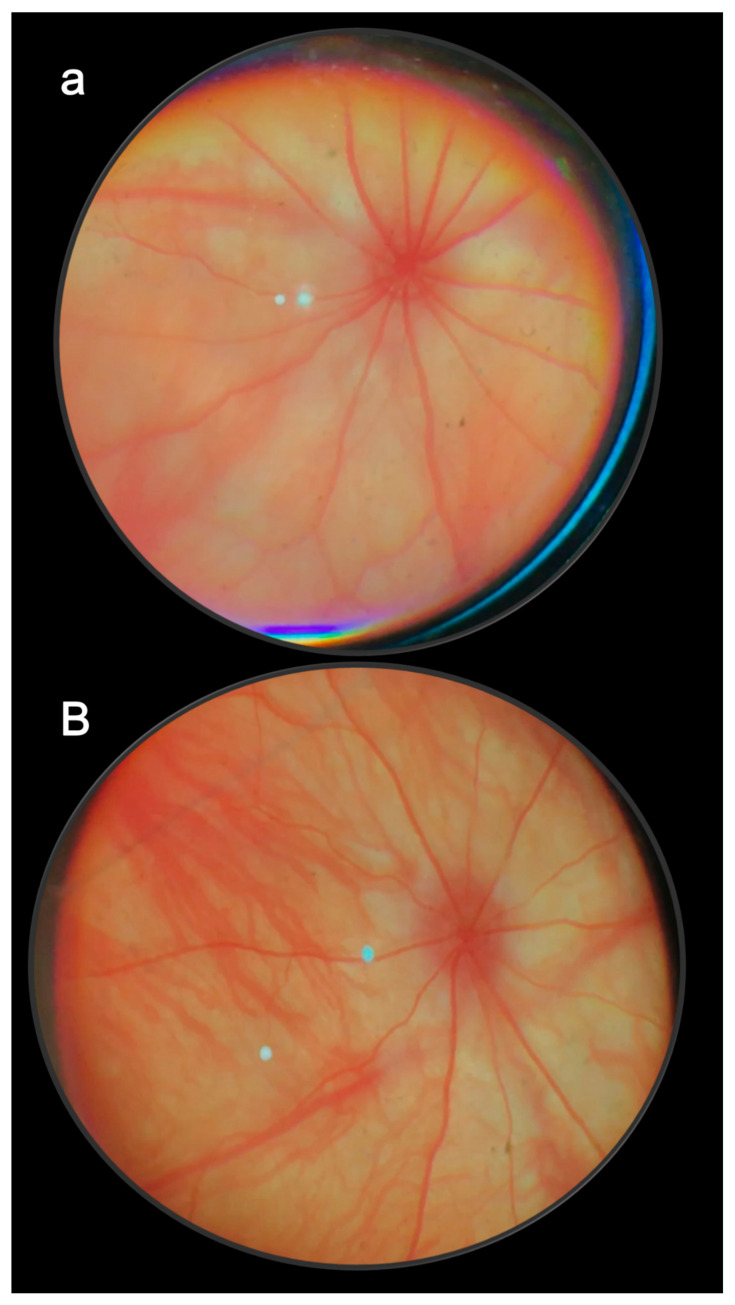
Presentation at 6th week of glaucoma in rats, control (**a**) and BPC 157-treated rats (**B**) (fundoscopy). Regularly, all control rats presented with strongly generalized irregularity of the diameter of the blood vessels with severe atrophy of the optic disc with deep excavation, and barely visible (extremely faint presentation) choroidal blood vessels (bright fundus background color) at the end of the sixth post-injury week (**a**). Contrarily, consequent to the evidence that all BPC 157 regimens, both prophylactic regimen and later, curative regimen, strongly reversed the increased intraocular pressure and abrogated mydriasis, there was normal fundus presentation, the presentation of the normal retinal and choroidal blood vessel presentation, and normal optic nerve presentation at the end of the sixth post-operative week (**B**) [11].

**Figure 2:**
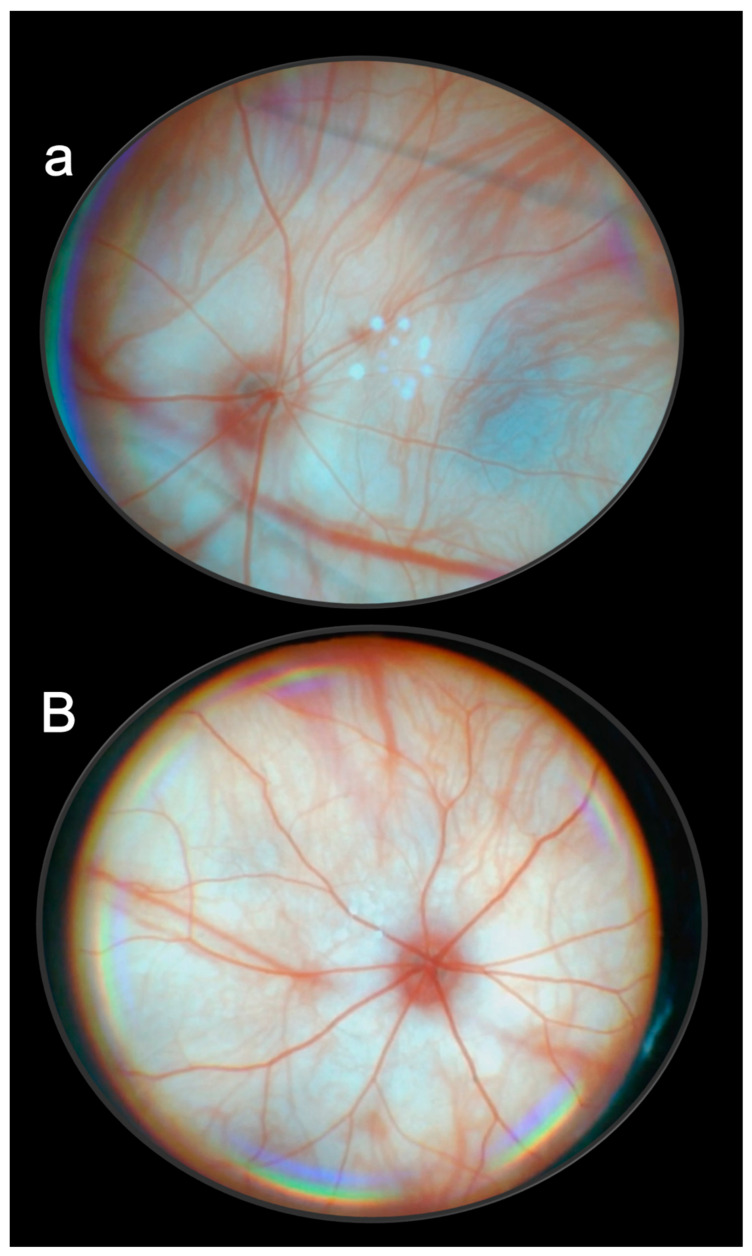
Presentation at 24 h of occlusion of infrarenal caval vein in rats, control (**a**), and in BPC 157-treated rats (**B**) (fundoscopy). Control rats presented with optic disc pallor with a very low filling of the arteries, and a huge veins/arteries ratio. Background choroidal circulation appearance suggests disturbance in circulation, respectively, decreased blood flow (**a**). Contrarily, there was a normal optic disc with the retinal arteries and veins in BPC 157-treated rats. Background choroidal appearance suggests circulation (**B**) [23].

These data are illustrated in Figure 1 (glaucoma rats) and Figure 2 (occluded infrarenal inferior caval vein, occlusion/occlusion-like syndrome).

## 3. Pupil Control

We specially reviewed the issue of BPC 157/NO-system very recently [97]. As a particular advantage, we used a “triple regimen” to envisage the NO-system as a whole. L-NAME/L-arginine/L-NAME + L-arginine, NO-system inhibition, NO-system over-stimulation, and NO-system immobilization were simultaneously tested, and thereby, interrelated [97]. BPC 157 application was given along with the application of L-NAME, along with the application of L-arginine, and along with the application of the combined L-NAME and L-arginine. In a large scale of distinctive targets, always using a “triple regimen”, it was possible to identify the particular effect of NOS-inhibition (L-NAME), the particular effect of NOS-over-stimulation (L-arginine), and the particular effect of NO-system immobilization (use of both NOS-antagonist and NOS-agonist together). Depending on the chosen target(s), the review analyzed more than 80 distinctive targets, which were used in previous NO-agents studies with “triple regimen”, and the particular effectiveness of the given NO-agent(s) (aggravation (mostly L-NAME), protection (mostly L-arginine), and no effect (either L-NAME alone or L-arginine alone, or both, or combination L-NAME + L-arginine) [97]. These revealed particular distinctive relations between the activities and inactivity of NO agents. These activities may be either protective or aggravating; NO-agents may act in an opposing way (L-NAME vs. L-arginine) but also in the same way, exhibiting a parallel effect (L-NAME ≈ L-arginine) [97]. Whatever is usually opposite or parallel as an exception (but quite often noted), NO-agents L-NAME and L-arginine activities may oppose each other’s effect (being NO-system related). Alternatively, they may not oppose each other’s effect (being NO-system non-related) [97]. Likewise, based on its interplay with L-NAME, L-arginine, and their combination, each of the investigated targets was defined as related to NOS-blockade or to NOS-stimulation or NO-system related and not related [97]. Thus, each target may produce a unique pattern of the NO-agents’ relations, and its significance and relation to other targets may be fully approached within the complexity of the NO-system functions. Note, a “triple regimen” (NO-system inhibition, over-stimulation, and immobilization simultaneously tested under the same experimental conditions) may reveal the particular complexity of NO-system functioning when the NO-system as a whole was investigated. As a prevailing pitfall, and unsuited over-simplification, such a NO-system complexity in functioning remained hidden in most studies using only one part regularly, mostly the NO-system inhibition as a theoretical background [97]. Thus, unlike “single-treatment”, which may only very partially depict possible NO-system involvement, BPC 157, known to induce NO-release by itself [98,99], is always investigated under the condition of the “triple regimen”, and may equally overwhelm the adverse effects of L-NAME, L-arginine, and their combination as well [97].

As indicated above, the BPC 157/NO-system may have special relation to pupil functioning [11,12]. This may be a special NO-system-related effect, namely, the parallel effect [96,97]. L-NAME, NOS-blockade, and L-arginine, NOS-substrate, with normal pupil, given either locally or systemically, produced prolonged miosis. They may antagonize each other’s effect when given together [12]. With atropine, both L-NAME, NOS-blockade, and L-arginine, NOS-substrate, antagonize atropine-mydriasis. However, in atropine-rats, unlike the mutually counteracting effect on miosis, they could not antagonize each other’s effect [12].

Consistently with the noted effect in rats with cauterized episcleral veins and glaucoma, BPC 157 without affecting the normal pupil, counteracted miosis produced by L-NAME, and miosis produced by L-arginine, as well as atropine-induced mydriasis [12]. Indicatively, BPC 157 counteracted atropine-mydriasis, but it did not counteract the counteraction effect of L-NAME or L-arginine on atropine-mydriasis. Similar effects appeared in rats and guinea pigs. Thus, in general, considering the known essential importance of the NO-system in eye functions [94,132,133,134,135,136], the evidence that BPC 157 therapy may cover both pharmacologically distinct NO-mechanisms (L-NAME vs. L-arginine) may be important. In pupil function regulation, these distinct NO-mechanisms (i.e., opposite effects on the same signaling pathway) produced the same physiological response [12]. Evidently, BPC 157 acted with each of them in order to achieve a normal pupil diameter again and counteract failed pupil function that may otherwise appear with atropine application [12].

Of note, this BPC 157 effect may be indicative of its particular anti-glaucomatous effect. Additionally, in support, BPC 157 may have a special effect on smooth muscle function, and ascertain the function of various sphincters as well [2,6,10].

**Figure 3:**
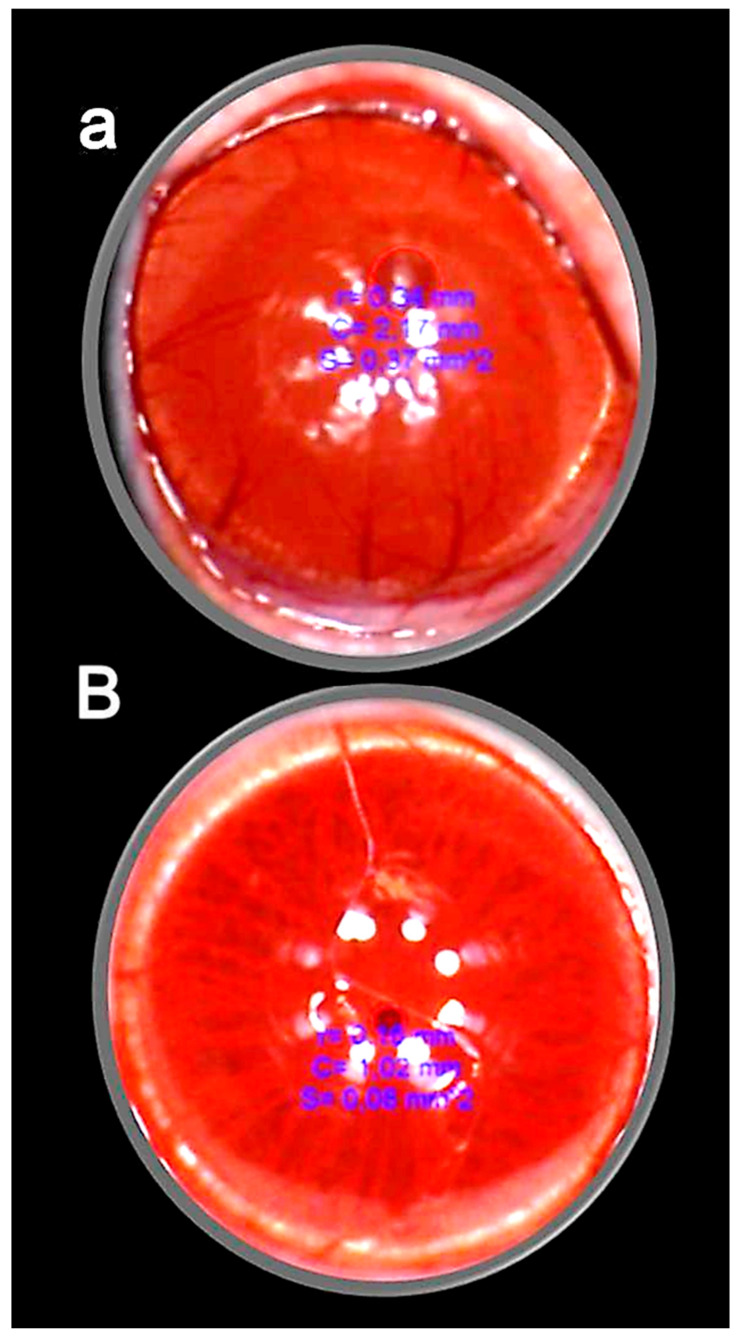
Persistent mydriasis in the atropine-induced mydriasis in rats after atropine administration (two drops of 1% atropine/eye) at 48 h in the control rats (**a**) (red circle), full counteraction of mydriasis and normal pupil presentation in BPC 157 treated rats (**B**) (small red circle). A similar effect was noted with all BPC 157 regimens. Veho Discovery VMS-004 Deluxe USB microscope camera [12].

These data are illustrated in Figure 3.

## 4. Retinal Ischemia

The evidence that BPC 157 counteracted retinal ischemia induced by NOS blocker L-NAME should be reviewed along with the general evidence of L-NAME vs. BPC 157 therapy [96,97,98,99,100]. This may be a direct cause–consequence noxious course relation and direct cause–consequence therapy relation. This may be particularly significant considering the essential physiological role of the NO-system [94,132,133,134,135,136]. Following the retrobulbar application of L-NAME, the retinal ischemia and definitively debilitated function occurred as an immediate and direct consequence of its inhibition. Likewise, the counteraction occurred as an immediate and direct consequence of the reversal of dysfunction. Thus, as we suggested, there may be direct relations. Direct (retrobulbar) L-NAME (one single) application goes toward breakdown of the NO-system (i.e., immediately produced generalized changes). Contrarily, direct (retrobulbar) BPC 157 application goes toward immediately reversed NO-system failure and recovered retinal function [13]. These findings of counteracted retinal ischemia should be overseen in the function of the particular anti-glaucomatous effect of BPC 157 therapy [11,12]. A particular controlling effect on pupil function was already claimed. These were based on the counteracted glaucoma-mydriasis [11], counteracted atropine-mydriasis [12], counteracted effect of NOS-blockade, counteracted L-NAME-miosis, counteraction of NO-system over-stimulation, and counteracted L-arginine-miosis [12].

Thereby, methodologically, the harmful effect of retrobulbar L-NAME application and the therapeutic effect of BPC 157 can both be direct effects with long-term consequences [13]. In such a way, defined direct effect may better serve the ischemia purpose than complex procedures with L-NAME. These were infusion into the anterior chamber, intraocular pressure above systolic pressure, and ligating the optic nerve [174,175,176,177,178,179]. However, unlike the consistent long-term effect induced by L-NAME retrobulbar application, there was only limited detrimental retinal effect at 40 min after intravitreous L-NAME application in cats lasting only for 180 min [179].

The stable pentadecapeptide BPC 157 therapy of retinal ischemia used all methodological advantages that might arise from the NOS blockade by L-NAME. One single retrobulbar application (5 mg/kg; 0.5 mg/0.1 mL saline/each eye) in a 4-week study permits a very simple and effective protocol, fundoscopy, behavior presentation, tonometry, and histology, to show timely progression of rapid retinal ischemia in rats [13]. Immediate lesions and subsequent rapid worsening progression to the severe stage was observed [13]. There was an immediate moderate generalized irregularity in the diameter of blood vessels with moderate atrophy of the optic disc and faint presentation of the choroidal blood vessels. Soon, there were generalized strong irregularity diameter blood vessels with severe atrophy of the optic disc and extremely poor presentation of the choroidal blood vessels. After one week, microscopy showed degeneration of ganglion cells, and nerve cell layer narrowing of the blood vessel lumen (using immunohistochemistry for factor VIII); particularly, damaged inner plexiform and inner nuclear layer with decreased thickness, along with complete retinal damage and decreased thickness. More and more damage occurred toward the end of the 2 and 4 weeks [13]. Importantly, there was function failure. Macro/microscopic failure was rapidly translated into a “frozen” behavior, with limited movements only, and an almost “frozen” rat, standing firmly on the surface with its posterior legs.

In principle, the failed presentation after retrobulbar L-NAME injection rapidly disappears upon the given BPC 157 therapy (retrobulbar application, 1 µg; 1 ng/0.1 mL saline/each eye, either 20 min or 48 h after L-NAME), which may characterize the counteracting potential of BPC 157 therapy [13]. This meant that there was a large therapeutic window, and a wide range of the agent’s efficacy (µg–ng), at any stage of the noxious course. Counteracting further development (i.e., 20 min after L-NAME) coincided with the reversal of the already advanced course (curative application at 48 h after L-NAME). Furthermore, histology assessment at 1, 2, and 4 weeks verified the counteraction of the damage of the inner plexiform layer and inner nuclear layer and revealed normal retinal thickness. The poor behavioral presentation was also rescued [13].

Noteworthy, there was normal intraocular pressure. However, initially, this model of the retrobulbar application implies a considerable but short-lasting increase in the intraocular pressure occurring in all rats. On the one hand, this would exclude the possible nonspecific contribution of the additional volume (applied to induce ischemia [174,175,176,177,178,179]). On the other hand, a considerable although short-lasting increase in intraocular pressure may trigger or contribute to the major noxious process seen to progress with the L-NAME retrobulbar application and the supposed failure of NO-system adaptive capabilities [13]. Contrarily, in BPC 157-treated rats, an otherwise insurmountable regular retrobulbar L-NAME injection course did not occur even during increased intraocular pressure or thereafter. By doing so, BPC 157 therapy may rapidly act to maintain retinal integrity. This implies sustaining increased intraocular pressure without harm or harmful consequences [13]. Similarly, sustaining the increased pressure within the body cavity without major harm also occurred in occlusion/occlusion-like syndromes [18,19,20,21,22,23,24,25,26,27,28,29,30]. Intracranial (superior sagittal sinus), portal and caval hypertension, aortal hypotension, ECG disturbances, and arterial and venous thrombosis, peripherally and centrally were counteracted. Lesions in the brain, heart, lung, liver, kidney, and gastrointestinal tract were counteracted. These were all ascribed to the essential reversal of the vascular and multiorgan failure and Virchow triad circumstances in the rat. Note, the occlusion/occlusion-like syndromes [18,19,20,21,22,23,24,25,26,27,28,29,30] include, permanent major vessel occlusion or permanent compression. An illustrative example may be sustaining the mechanically maintained high intra-abdominal hypertension, 25 mmHg, 30 mmHg, 40 mmHg, and 50 mmHg, for a considerable time. BPC 157 counteracted the consequences of grade III and grade IV intra-abdominal hypertension [29]. It was demonstrated that the recovery is due to BPC 157-activated azygos vein direct blood flow delivery serving as an upgraded minor vessel that may quickly act for the failed (compressed) major vessel, reestablishing the reorganized blood flow [18,19,20,21,22,23,24,25,26,27,28,29,30]. A specific analogy may be envisaged for retrobulbar L-NAME-induced retinal ischemia. The damaged inner plexiform layer and inner nuclear layer may be specifically targeted as they are supplied through the central retinal artery [180]. This can directly verify the central retinal artery affected by L-NAME (i.e., vasoconstriction), with adequate time to produce/initiate the chain of damaging events, further lesion progress, and progressive NO-system failure from week 1 to week 2 to week 4 in L-NAME-treated rats [13]. Contrarily, the results of the given BPC 157 therapy are the counteraction of the damage to the inner plexiform layer and inner nuclear layer as well as the revealed normal retinal thickness. This verifies the concept as valuable for the therapy against L-NAME, NO-system failure, and retinal ischemia. Thereby, the recovery of the central retinal artery may be a particular target recovery realized by applying BPC 157 therapy [13].

Finally, in general, prompt counteraction of L-NAME retinal ischemia complies with the consistent counteraction by BPC 157 therapy application to the various L-NAME-induced damaging effects in different models and species [96,97].

**Figure 4:**
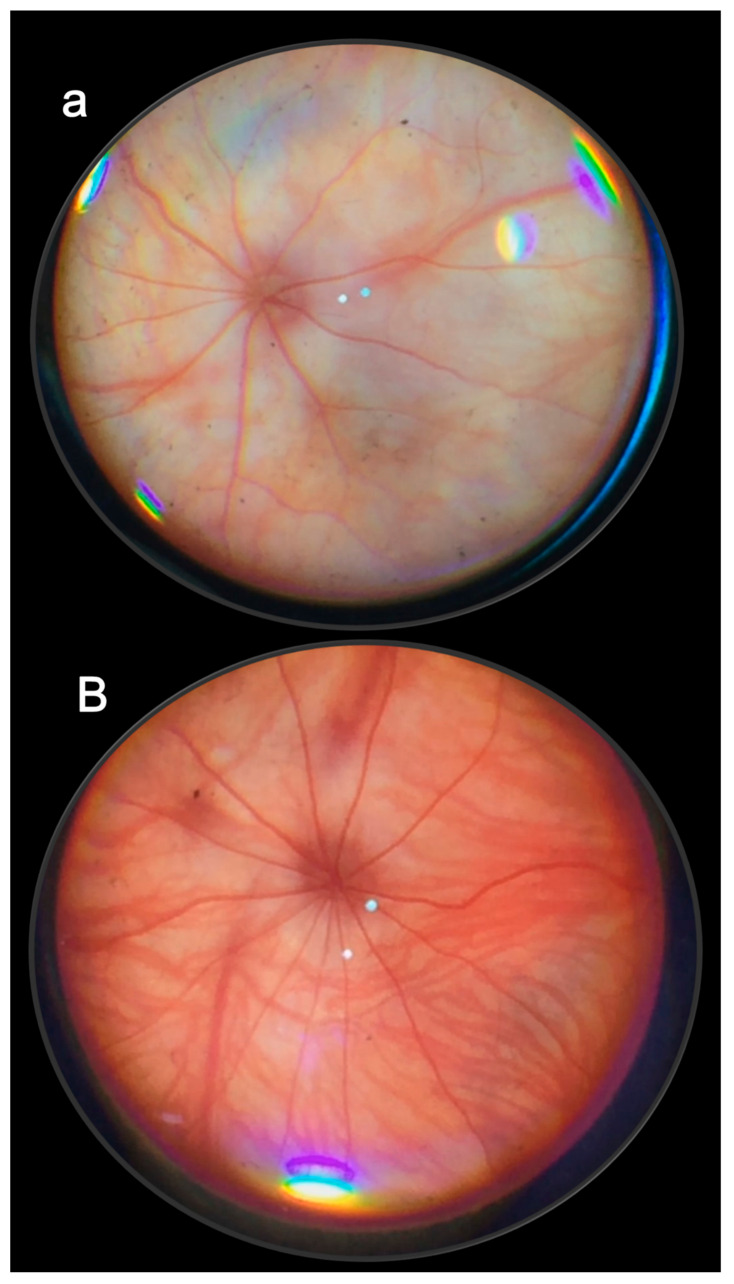
Presentation at one week after retrobulbar application of L-NAME in rats, control (**a**), and in BPC 157-treated rats (**B**) (fundoscopy). All control rats presented with strong generalized irregular diameter blood vessels with severe atrophy of the optic disc, and extremely poor presentation of the choroidal blood vessels at the end of week 1 (**a**). Contrarily, all BPC 157-treated rats were presented with normal eye backgrounds and normal presentation of the retinal and choroidal blood vessels at the end of week 1 (**B**) [14].

These data are illustrated in Figure 4.

## 5. Cornea

We evidenced that BPC 157 therapy cured severe corneal lesions and maintained corneal transparency. This was held as a promising part of the particular vascular function recovery in glaucoma and retina ischemia and control of pupil function [11,12,13]. The therapy regimen was 2 pg/mL, 2 ng/mL, and 2 µg/mL distilled water, two eye drops/left rat eye immediately after injury induction, and then every 8 h up to 120 h [14]. The equipotent therapy range successfully closed perforating corneal incisions in rats and rapidly recovered corneal transparency. Likewise, after total debridement of corneal epithelium and completely denudated cornea, it accelerated corneal recovery and maintained corneal transparency [14,15]. After lacrimal gland extirpation, BPC 157 counteracted the damaging effects of dry eye syndrome in rats [16,17].

First, in general terms, this may be important. Namely, maintained (recovered) corneal transparency (and thereby maintenance of visual function) implies that the therapy may successfully recover the integrity of all its components (i.e., epithelium, stroma, and endothelium) [181,182]. Moreover, in general terms, the prevented/reversed corneal swelling should include the recovered metabolic and functioning as diffusion barriers to the fluid (tears or aqueous humor) of the epithelium and endothelium, in particular [182,183,184,185,186]. This should be achieved given the major role of maintaining corneal hydration; the endothelium barrier and pump functions should both be recovered to achieve the recovery of corneal swelling and opacity [182,183,184,185,186]. Noteworthy, BPC 157 therapy commences after cornea perforation. Thereby, it encompasses rapid recovery of the damaged endothelium toward an endothelial barrier with intact tight junctions. That may function continually for the proper functioning of the pump–leak system [182]. This may be important since BPC 157, as mentioned, acts as a membrane stabilizer, recovering leaky gut syndrome. It acts via increasing tight junction protein ZO-1 expression and transepithelial resistance [107]. Contrarily, the damaged endothelium is known to regularly only have little regenerative capacity [181,187].

Second, there were regularly developed new vessels presented from the limbus to the penetrated area in control rats after cornea perforation and loss of transparency [14]. Consequently, BPC 157 therapy, corneal transparency such as in the normal avascular cornea, evidently resolved cornea “angiogenic privilege” known to be essential for cornea healing and transparency maintenance [188,189,190,191]. Thereby, BPC 157 effects envisage a balance between competing proangiogenic and antiangiogenic mediators. Corneal neovascularization was strongly counteracted whatever the cause. In principle, this might be inflammation, the invasion of endothelial cells into the cornea during inflammation largely stimulated by the actions of macrophages. Likewise, corneal neovascularization might be induced by hypoxic conditions (upregulation of proangiogenic factors, downregulation of anti-anxiogenic factors to supply oxygen to the cornea) [181]. Note, in BPC 157-treated rats, an ameliorated healing course occurred with apparently less aqueous cells [14] and BPC 157 may particularly modulate vascular endothelial growth factor (VEGF) activities [104,192,193] essential for cornea neovascularization.

Thereby, the findings that BPC 157-treated rats generally had no new vessels, and those that did form in the limbus did not make contact with the penetrated area [14,15], which was consistent with evidence. As such, it means essential points are fully resolved for both corneal wound healing and wound healing in general and applied BPC 157 therapy [192,193]. Most importantly, these were in accordance with the identified particular vascular target for successful therapy in the rat glaucoma, retinal ischemia, and corneal ulcers (i.e., episcleral veins, central retinal artery). There may be a blood supply to the cornea. There are tiny vessels at the outer edge of the cornea as well as components supplied by end branches of the facial and ophthalmic arteries through the aqueous humor and the tear film [180]. Finally, the rapid regaining of corneal transparency illustrates the modulated tissue-specific healing effects of the BPC 157 therapy effects well (cornea vs. other tissues). A strong angiogenic effect was wound healing angiogenesis in the BPC 157 therapy of other tissues [2,6,192,193]. This was evidenced as a part of the advanced healing [2,6,192,193] (note, in hind ischemia recovery, BPC 157 accelerates the blood flow recovery and vessel number) [104], including other avascular tissues [2,6,192,193]; an example of which is tendon healing. In particular, BPC 157 therapy resolved several aspects of tendon healing. The healing of transected tendon, osteotendinous junction following Achilles’ tendon detachment, and myotendinous junction were reported [2,6,10]. BPC 157 might modulate VEGF expression, whereas in vitro, BPC 157, unlike VEGF, does not stimulate angiogenesis [2,6,192,193] as an important part of the particular local and systemic wound healing effects (for review see [1,2,3,4,5,6,7,8,9,10]).

**Figure 5:**
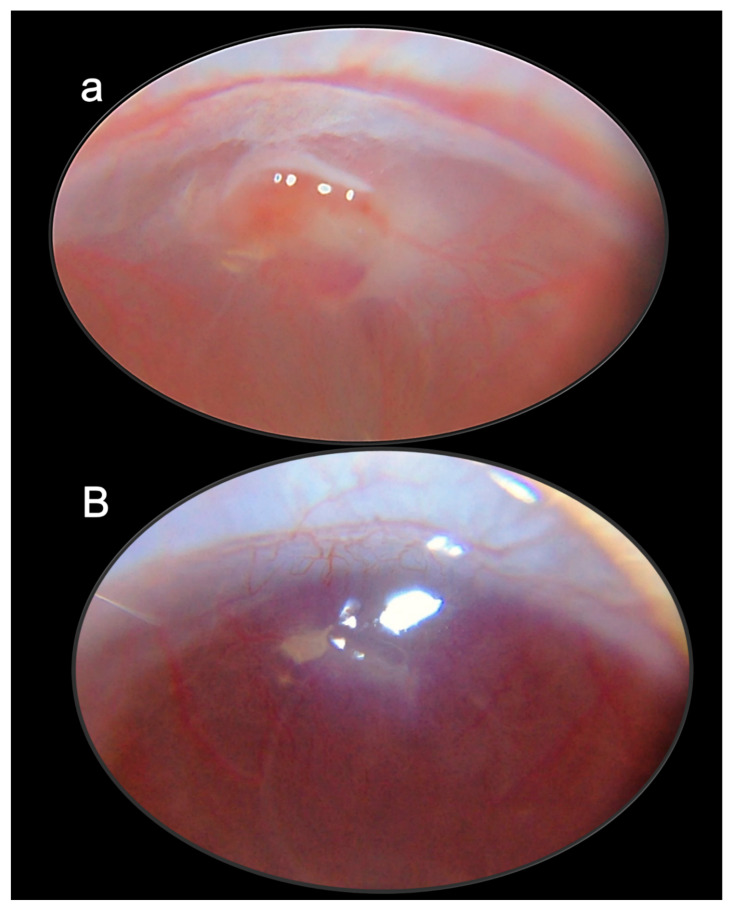
Corneal perforating injury. Characteristic gross presentation at 72 h post-surgery in controls (**a**), and BPC 157-treated rats (**B**); 60× magnification. Regularly, controls presented with edema at the site of ulceration, growth of new vessels, corneal opacity, and poor transparency. The inflammatory process is active (**a**). Contrarily, BPC 157-treated rats exhibited an absence of edema at the site of ulceration, markedly attenuated new vessels, corneal transparency, and no signs of inflammatory process at day 3 (**B**) [15].

**Figure 6:**
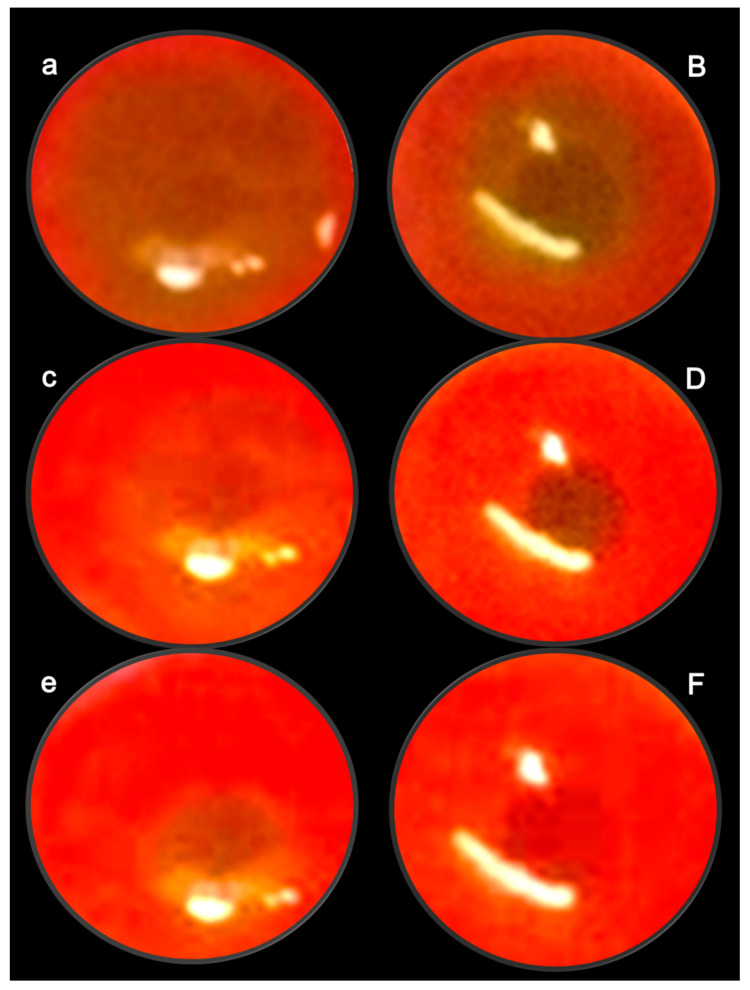
Characteristic eye presentation in control rats (lowercase letters), and in BPC 157-treated rats (capitals) after entire epithelium removal, at 16 h (**a**,**B**), 24 h (**c**,**D**), and 48 h (**e**,**F**). The defect area was stained green by fluorescein. Characteristic healing acceleration toward the corneal surface completely healed in the BPC 157-treated eye [14].

These data are illustrated in Figure 5 and Figure 6.

## 6. Restoration of Corneal Sensitivity

We interpreted the obtained BPC 157 therapy evidence in the recovery of tetracaine-, oxybuprocaine-rats [17] in relation to recovered corneal particularities (i.e., profound innervation and extensive sensitivity) [180]. BPC 157 shortens corneal anesthesia [17], counteracts the lesions, and recovers the tear volume and blinking. In support, BPC 157 counteracts the damaging effects of lacrimal gland extirpation and dry eye syndrome in rats [16]. Thereby, restoration of corneal sensitivity occurred as a consistent outcome of the BPC 157 eye therapy. This may reveal the suggested particular vascular function recovery in glaucoma and retina ischemia, pupil function controlling (counteracted glaucoma-mydriasis, atropine-mydriasis, and NO-agents-miosis) [11,12,13], and healing of severe corneal lesions and recovering corneal transparency (i.e., corneal ulcer, complete denudation of cornea) [14,15]. In this chain of noxious events following tetracaine-, oxybuprocaine-corneal anesthesia, BPC 157 therapy effects may have a particular interaction with the NO-system. There was a counteraction of L-NAME worsening and upgrading of otherwise L-arginine-limited therapeutic effect [17].

This may be a particular chain of events achieved with BPC 157 therapy, in particular. Namely, decreased corneal sensitivity and tear formation appeared with many standard anti-glaucoma agents (i.e., latanoprost, timolol, brimonidine, and dorzolamide) [194,195,196,197]. Thus, these may further underscore the particular relevance of the BPC 157 anti-glaucomatous effect [11,12,13,14,15,16,17], as well as in a particular relation with the NO-system, as mentioned above. In general, given the distinctive effects of NO-agents L-NAME and/or L-arginine in the eye studies (i.e., retinal ischemia, pupil control, corneal anesthesia) [12,13,17], few distinctive NO-pathways might be correspondingly presented [96,97]. The counteraction potential of BPC 157 therapy suggests that BPC 157 in eye therapy may successfully interact with these distinctive NO-pathways [96,97]. Thereby, it may counteract corresponding disturbances, namely, those related to NO-system blockade (i.e., retinal ischemia [13], prolonged miosis [12], and prolonged corneal insensitivity [17]). Likewise, it may counteract those combined with NO-system over-stimulation (i.e., prolonged miosis) [12]. Thus, as mentioned before, this may indicate its modulatory potential for the eye NO-system, where maintained undisturbed eye NO-system function is essential. Namely, NO-inhibition (LNAME-induced retinal ischemia) [13] and NO-over-stimulation (overproduction of NO interacting with oxygen radicals induced the death of retinal neurons) [198,199,200] may be both harmful. BPC 157 may consistently antagonize the adverse effects of NO-system inhibition as well as the adverse effects of NO-system overstimulation, presented in the eye as well as in other body systems [12,13,17,96,97].

Second, with local anesthetics, a particular chain of events can be envisaged [17] in the corneal anesthesia counteraction and counteraction of the effects of different classes of local anesthetics. Counteracted corneal insensitivity following local eye application [17] occurred along with counteraction of the local anesthetic’s effects of intraplantar application, axillary block, spinal block, arrhythmias, and seizures [201,202]. Thereby, consistent counteraction by BPC 157 therapy, intraplantar, intraperitoneal, and intragastric application, and local eye drops, may suggest interference with particular targets commonly known for local anesthetic effects. Note, BPC 157 therapy might have a direct effect on observable potassium conductance in HEK293 cells. There was in vitro (HEK293 cells) inhibition of lidocaine- [201], bupivacaine- [202], hypermagnesemia- [203], hyperkalemia- [204] induced depolarization by BPC 157. Indicatively, BPC 157 also abolished hyperpolarizations of HEK293 cells during hypokalemic conditions [205]. Thus, the investigated topical ophthalmic anesthetics applied onto the surface of the eye and known to act by blocking sodium channels in neuronal axons [206], may suggest an additional particular target for BPC 157 therapy, ascertaining conduction along the axons and keeping the eye–brain functioning continuously. In such consideration [207], along with counteraction of the damaging effects of lacrimal gland extirpation and dry eye syndrome in rats [17], counteracting topical ophthalmic anesthetic inhibition, with the eye open, the light-evoked increases in tear volume, evidenced that BPC 157 can permanently evoke protective reflexes [17].

**Figure 7:**
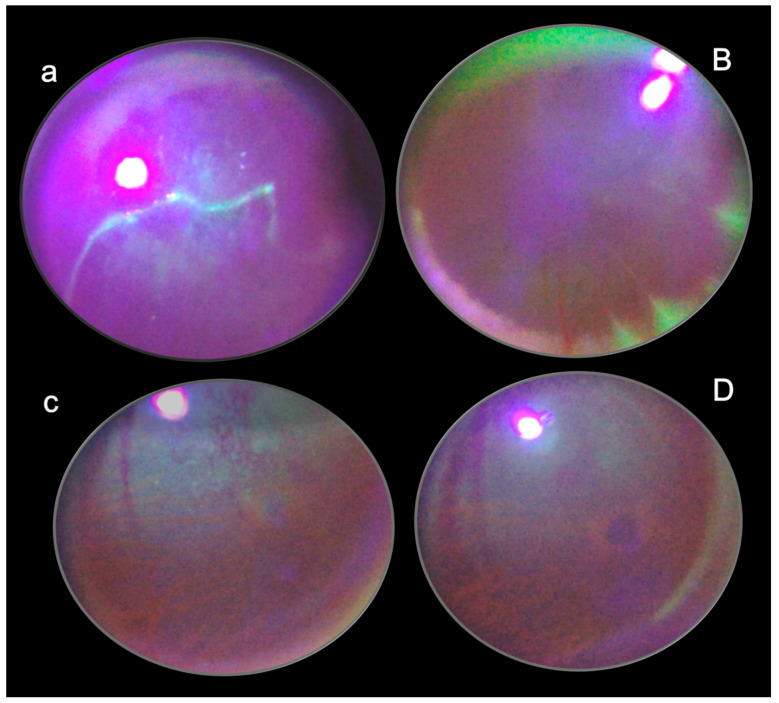
Characteristic eye presentation in control rats (lowercase letters), and in BPC 157-treated rats (capitals) after lacrimal gland removal, at 150 min (**a**,**B**) and 6 weeks (**c**,**D**). In controls, staining the cornea with fluorescein dye and examination under blue light shows positive fluorescein test, and epithelial defects at 150 min (**a**) and 6 weeks (**c**). Contrarily, in BPC 157-treated rats, staining cornea with fluorescein dye and examination under blue light shows negative fluorescein test, and absence of the epithelial defects at 150 min (**B**) and 6 weeks (**D**) [17].

These data are illustrated in Figure 7.

## 7. Conclusions

### 7.1. Cytoprotection Concept in Eye Pharmacotherapy

In conclusion, we envisaged all elaborated eye lesions [11,12,13,14,15,16,17] as direct damage, and prototype lesions that as such should be the core hallmark in cytoprotection studies [42,46]. These were cauterization of episcleral veins to produce glaucoma, atropine and NO-agents application, perforated cornea, corneal abrasion, corneal anesthesia, and lacrimal gland extirpation. This conceptual approach might be further indicative of additional implementation of the cytoprotection concept into eye pharmacotherapy and the whole occlusion/occlusion-like syndrome [18,19,20,21,22,23,24,25,26,27,28,29,30]. Likewise, it may be indicative of a demonstration of the therapeutic potential of the stable gastric pentadecapeptide BPC 157, in particular, and cytoprotective agents in general [1,2,3,4,5,6,7,8,9,10]. There was the rapid recruitment of the activated collateral pathways, and the activated upgraded minor vessel becomes capable of substituting the disabled function of the failed major vessel, and reestablishing reorganized blood flow [18,19,20,21,22,23,24,25,26,27,28,29,30]. In eye pharmacology [11,12,13,14,15,16,17], this novel therapeutic approach with the stable gastric pentadecapeptide BPC 157 means that after the cauterization of the episcleral veins [11], one remaining episcleral vein takes over the function of all (four) episcleral veins, and recovers adequate functioning. These appeared as having modulatory effects on the important systems, essentially implicated in cytoprotection [96,97,106], and eye functioning [94,95], NO-system, and prostaglandins systems [94,95,96,97,106]. BPC 157 therapy involves the NO-system as a whole (NO-release, NOS-inhibition, NO-over-stimulation, all affected) [96,97,98,99,100], controlling vasomotor tone, and the activation of the Src-Caveoli-1-eNOS pathway [103,104]. Likewise, there is the modulatory effect on the prostaglandin systems. BPC 157 therapy counteracted NSAID toxicity, counteracted bleeding thrombocytopenia, and, in particular, leaky gut syndrome [106,107]. Thus, it might be a cytoprotection phenomenon with application in distinctive injuries and circumstances (the normalization of increased intraocular pressure, recovery of glaucoma along with the recovery of pupil function, retinal ischemia, and corneal sensitivity and lesion and maintained corneal transparency) [11,12,13,14,15,16,17]. It should be analogous to, if not identical to, that which might occur during major vessel occlusion and application of the other similar noxious procedures [18,19,20,21,22,23,24,25,26,27,28,29,30]. Furthermore, it might be that in both cases, the same agent—pentadecapeptide BPC 157, also suggested to be a novel cytoprotection mediator as it is native and stable in human gastric juice, and easily applicable (locally, eye drops and systemically, including via per-oral way) [1,2,3,4,5,6,7,8,9,10,96,97,106,107,193]—might be responsible for the effect.

### 7.2. Correlation between Glaucomatous Rats and Occlusion/Occlusion-like Syndromes Rats as Cytoprotection Implementation

Thus, this principle [1,2,3,4,5,6,7,8,9,10,96,97,106,107,193] combined particular findings in investigated/counteracted glaucomatous rats [11] and those investigated/counteracted in the occlusion/occlusion-like syndromes [18,19,20,21,22,23,24,25,26,27,28,29,30]. Thus, there were increased intraocular pressure and mydriasis, degeneration of retinal ganglion cells, optic nerve head excavation and reduction in optic nerve thickness, generalized severe irregularity of retinal vessels, faint presentation of choroidal vessels, and severe optic nerve disc atrophy in glaucomatous rats [11]. These may correspond to the intracranial (superior sagittal sinus), portal, and caval hypertension, aortal hypotension, ECG disturbances, brain swelling and lesions, heart dysfunction, lung lesions, liver, and kidney failure, gastrointestinal lesions, widespread arterial and venous thrombosis, venous congestion (i.e., inferior caval vein and superior mesenteric vein), and venous failure (azygos vein) in the occlusion/occlusion-like syndromes [18,19,20,21,22,23,24,25,26,27,28,29,30]. As pointed out, these were all attenuated/eliminated by the similar BPC 157 regimens and similar effect (i.e., activation of the collateral circulation) and might provide full support [18,19,20,21,22,23,24,25,26,27,28,29,30]. The most important point is that in glaucomatous rats with high intraocular pressure (three of four episcleral veins cauterized), all BPC 157 regimens rapidly normalized intraocular pressure and one remaining episcleral vein, as upgraded, worked for overall function [11]. BPC 157-treated rats exhibited normal pupil diameter, microscopically well-preserved ganglion cells and optic nerve presentation, normal fundus presentation, normal retinal and choroidal blood vessel presentation, and normal optic nerve presentation [11]. In glaucoma studies such as in in other eye disturbances studies (i.e., retinal ischemia), BPC 157 therapy may instantly break a noxious chain of events, both at its early stage and at an already advanced stage [11,12,13,14,15,16,17].

**Figure 8:**
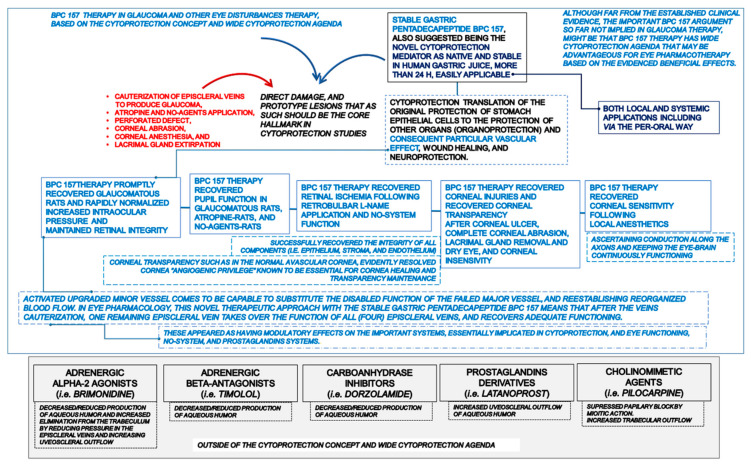
Though having a wide range of structures and targets, glaucoma therapy (red marks), BPC 157 therapy effects are united by their ability to produce marked definitive cytoprotective effects, as seen with evidenced general recovery in glaucomatous rats (blue marks). There was a shared ability to recover glaucomatous rats [11] and rapidly normalize intraocular pressure [11], maintain retinal integrity [11], recover pupil function [11,12], recover retinal ischemia [13], recover corneal injuries, recover corneal transparency, and regain corneal sensitivity [14,15,16,17]. Recovered corneal injuries and recovered corneal transparency were exemplified in rats who underwent complete corneal abrasion [14], corneal ulceration [15], lacrimal gland removal dry eye, or corneal insensitivity [16,17]. Contrarily, targets of standard intraocular pressure-lowering drugs [30,31,32,33,34] such as reducing the production of aqueous humor (i.e., beta-blockers, alpha 2-agonists, inhibitors of carbonic anhydrase), promoting drainage through the trabecular meshwork (parasympathomimetics), and through uveoscleral route (prostaglandin derivatives), all remained outside of the cytoprotection implementation [30,31,32,33,34] (gray area). A common concern given the potential impact of the standard intraocular pressure lowering agents on the corneal endothelium is observable [74,75]. Beta-blockers [76,77,78], alpha2-adrenoreceptor agonists [79,80,81], carbonic anhydrase inhibitors [82,83,84,85], and prostaglandin analogs [86,87,88,89] were mentioned. In addition, latanoprost caused miosis, with rebound mydriasis at 24 h post-treatment [150]. Timolol has a delayed effect on miosis (from 4 to 8 h post-treatment), as well as in normal eyes [151,152]. Dorzolamide had no effect [153]. Pilocarpine induced 30 min–12 h miosis in normal eyes [154]. Finally, decreased corneal sensitivity and tear formation appeared with many standard anti-glaucoma agents (i.e., latanoprost, timolol, brimonidine, and dorzolamide) [194,195,196,197]. Note, the standard anti-glaucomatous agents on the increased intraocular pressure had delayed onset [155,156,157,158,159,160,161,162], and in some experimental studies, the effect can be absent [163], or even have the opposite effect [164].

Thus, the cytoprotective wide principle of the possible BPC 157 therapy in glaucoma and other eye disturbances [11,12,13,14,15,16,17] may likely be distinctive and more productive than those commonly known for standard intraocular pressure-lowering drugs [30,31,32,33,34]. It may be that reducing the production of aqueous humor (i.e., beta-blockers, alpha 2-agonists, inhibitors of carbonic anhydrase), promoting drainage through the trabecular meshwork (parasympathomimetics), and through the uveoscleral route (prostaglandin derivatives). they all remained outside of the cytoprotection implementation [30,31,32,33,34]. This was summarized in Figure 8.

### 7.3. Possible Regulatory Physiologic Role in Eye and Bodily Functions for Clinical Safety and Efficacy as Concluding Remarks

Finally, BPC 157 was found in many tissues in both human fetuses and adults (i.e., gastrointestinal mucosa, lung bronchial epithelium, the epidermal layer of the skin, and kidney glomeruli) (i.e., in situ hybridization and immunostaining) [8,193]. Consequently, as might be evidenced from the presented anti-glaucomatous therapy and therapy of other eye disorders [11,12,13,14,15,16,17], parallel to resolving the complex occlusion/occlusion-like syndrome [18,19,20,21,22,23,24,25,26,27,28,29,30], BPC 157 may have a regulatory physiological role in eye and bodily functions. Based on similar beneficial effects, similar importance was also suggested for other species (i.e., birds [208] and insects [209,210]). This might coincide with a very safe BPC 157 toxicity profile. No adverse effects were noted in clinical trials (ulcerative colitis, phase II), and in toxicological studies, a lethal dose (LD1) could be not achieved (for review see [1,2,3,4,5,6,7,8,9,10,96,97,106,107,193]). This favorable point was recently confirmed in a large study conducted by Xu and collaborators [211]. Together, these findings and the wide range of effectiveness of the BPC 157 regimens (pg, ng, µg, eye drops, or systemically, including via per-oral route) (for review see [1,2,3,4,5,6,7,8,9,10,96,97,106,107,193]) may be suggestive of further BPC 157 therapy application. In general, in eye therapy in particular, the BPC 157 therapy may highlight its particular capabilities, cytoprotection [7] and particular vascular effect [1], wound healing [8], and neuroprotection [9].
